# Supplementary material for: Content-rich biological network constructed by mining PubMed abstracts
Source: BMC Bioinformatics. 2004 Oct 8;5:147. doi: 10.1186/1471-2105-5-147 (PMC528731; doi:10.1186/1471-2105-5-147)
Supplement: Additional File 5 — The original Chilibot query results of the term "long-term potentiation (LTP)" and 22 other terms, limiting the latest references analyzed to the years 1990, 1995, 2000, and 2004. [file 1471-2105-5-147-S5.bz2 › chilibotAdditionalFile5/ltp2000/html/PLC_TRKA.html]

 


 **PLC** and **TRKA** 
  
Found 15 abstracts in PubMed,  **15 abstracts were retrieved and analyzed**.  


---

 Search Google  |
 PDF files only 
|  EDU domain only 

---

**Interactive relationship** (e.g. stimulation, inhibition, etc)

- In contrast, the  **TrkA**  Y490F mutant receptor does not delay U.V. induced apoptosis whilst  **TrkA**  mutated at its  **PLC**  gamma binding site Y785F is capable of protecting from apoptosis induced by c Myc or U.V. treatment.  Ref: 9484773 Oncogene, 1998
- Nerve growth factor induces a multimeric  **TrkA**  receptor complex in neuronal cells that includes Crk, SHC and  **PLC**  gamma 1 but excludes P130CAS.  Ref: 8552402 Oncogene, 1996
- Both TrkC and  **TrkA**  mediate qualitatively similar increases in the tyrosine phosphorylation of phospholipase C  **PLC**  gamma1, Shc, SNT, and MAPK and the transcription of the c fos, c jun, NGFI A, and NGFI B immediate early genes.  Ref: 8621434 J Biol Chem, 1996
- Using receptor activated immunoprecipitation followed by analysis of the immune complexes by SDS PAGE and immunoblotting, we show that NGF induces the association of  **TrkA**  with c Crk II in a multimeric complex that also includes SHC and  **PLC**  gamma 1.  Ref: 8552402 Oncogene, 1996
- After NGF treatment, NGF was bound to  **TrkA**  in endocytic organelles, and  **TrkA**  was tyrosine phosphorylated and bound to  **PLC**  gamma1, suggesting that these receptors were competent to initiate signal transduction.  Ref: 8987823 J Neurosci, 1996
- Product and dead end inhibition studies indicate an ordered association of substrates to  **trkA**  kinase with the nucleotide ATP binding prior to the exogenous substrate  **PLC**  gamma GST, followed by release of the phosphorylated  **PLC**  gamma GST product prior to release of ADP sequential ordered bi bi mechanism.  Ref: 9448714 Arch Biochem Biophys, 1998

**Parallel relationship** (e.g. studied together, co-existance, homology, etc.)

- These results indicate that in developing cortical neurons, activation of AMPA KA receptors by KA may induce expression, followed by activation of  **TrkA**  via  **PLC**  signaling and intracellular calcium elevation and hence increase reception of NGF on KA challenged neurons.  Ref: 10820201 J Neurochem, 2000
- Unglycosylated  **TrkA**  core protein is phosphorylated even in the absence of ligand stimulation and displays constitutive kinase activity as well as constitutive interaction with the signaling molecules Shc and  **PLC**  gamma.  Ref: 10235685 J Neurobiol, 1999
- Mutational analysis indicates that coactivation of phospholipase C gamma  **PLC**  gamma and phosphoinositide 3 kinase PI3 kinase by specific cytoplasmic domains of  **TrkA**  is essential for triggering chemoattraction of the growth cone in an NGF gradient.  Ref: 10402200 Neuron, 1999
- While the tyrosine phosphorylation of  **TrkA** , SHC and  **PLC**  gamma 1 increased with time in the presence of NGF and was inhibited by the tyrosine kinase inhibitor K252a, the state of tyrosine phosphorylation of c Crk II did not appear to change with NGF treatment.  Ref: 8552402 Oncogene, 1996
- A study of polyphosphoinositide turnover showed that KA stimulated phospholipase C  **PLC**  activity was directly triggered by the AMPA KA receptor activity, but not by the activity of  **TrkA**  or other excitatory amino acid receptor subtypes.  Ref: 10820201 J Neurochem, 2000
